# Preparation and properties of copper-oil-based nanofluids

**DOI:** 10.1186/1556-276X-6-373

**Published:** 2011-05-05

**Authors:** Dan Li, Wenjie Xie, Wenjun Fang

**Affiliations:** 1 Department of Chemistry and Chemical Engineering, Weifang University, Weifang 261061, China; 2 Qianjiang College, Hangzhou Normal University, Hangzhou 310027, China; 3 Department of Chemistry, Zhejiang University, Hangzhou 310027, China

## Abstract

In this study, the lipophilic Cu nanoparticles were synthesized by surface modification method to improve their dispersion stability in hydrophobic organic media. The oil-based nanofluids were prepared with the lipophilic Cu nanoparticles. The transport properties, viscosity, and thermal conductivity of the nanofluids have been measured. The viscosities and thermal conductivities of the nanofluids with the surface-modified nanoparticles have higher values than the base fluids do. The composition has more significant effects on the thermal conductivity than on the viscosity. It is valuable to prepare an appropriate oil-based nanofluid for enhancing the heat-transfer capacity of a hydrophobic system. The effects of adding Cu nanoparticles on the thermal oxidation stability of the fluids were investigated by measuring the hydroperoxide concentration in the Cu/kerosene nanofluids. The hydroperoxide concentrations are observed to be clearly lower in the Cu nanofluids than in their base fluids. Appropriate amounts of metal nanoparticles added in a hydrocarbon fuel can enhance the thermal oxidation stability.

## Introduction

Nanofluid is a novel heat-transfer fluid prepared by dispersing nanometer-sized solid particles in traditional heat-transfer fluid to increase thermal conductivity and heat-transfer performance. Nanofluid was coined by Choi and colleagues [1-3] in 1995 at Argonne National Laboratory of the USA. Nanofluids with water, ethylene glycol, or oil as the base fluid were of great significance primarily because of their enhanced thermal properties. There are compelling needs in many industrial fields to develop oil-based heat transfer fluids with significantly higher thermal conductivity for energy-efficient heat exchangers. Many efforts have been focused on the oil-based nanofluids. Transformer oil, mineral oil, silicon oil, hydrocarbon fuels, and some organic solutions are used as the base fluids for studying nanofluids. The dispersion and thermal conductivities of the oil-based nanofluids containing Cu, CuO, Ag, or Al_2_O_3 _particles have been recently reported [4-6].

When nanoparticles are introduced into oil, the particles are usually sedimented within several minutes because of the poor compatibility between the nanoparticles and the base oil. The agglomerated particles are gradually settled over time, which leads to the poor stability and low heat-transfer capability of the suspensions. Thus, an appropriate lipophilic modification process is needed for the formation of a stable oil-based nanofluid. Surface modification on metallic particles with hydrophobic ligands and addition of dispersant can be employed to improve the compatibility between the nanoparticles and the oil-based fluid. The organic ligands with long hydrocarbon chains coordinated to the nanoparticles prevent the particles from clustering, and the surface-modified nanoparticles possess good dispersion behavior in oils [4,7-9].

Kerosene, a typical hydrocarbon fuel, circulated in aircraft for cooling can serve as the primary thermal sink by dissipating waste heat from aircraft subsystems. However, it has relatively low thermal conductivity. As is well known, a kerosene-based nanofluid can improve the heat transfer property and cooling capacity. In this study, we attempted to synthesize lipophilic Cu nanoparticles and to prepare oil-based nanofluids. The hydrophobic layers formed on the surface of copper nanoparticles can protect the particles against oxidation and improve dispersion stability of oil-based nanofluids [10-12], which are important for exploiting the potential benefits and applications of the enhanced thermal properties of the nanofluids. In the meanwhile, the effects of the lipophilic Cu nanoparticles on the viscosity, thermal conductivity, and thermal oxidation stability of the nanofluids are also investigated.

## Experimental

### Materials and preparation of ligand

All the materials and solvents used in this study, P_2_S_5_, cetyl alcohol, anhydrous ammonia, benzene, cupric acetate, ethanol, sodium hypophosphite, hydrazine hydrate solution (85%), toluene, decahydronaphthalene, and dichloromethane were analytic grade agents.

The Cu nanoparticles were prepared and modified by O, O-di-*n*-cetyldithiophosphoric acid. The O, O-di-*n*-cetyldithiophosphate [13] was synthesized by heating P_2_S_5 _(0.02 mol) and cetyl alcohol (0.07 mol) at 80°C for 3 h. The suspension was cooled to room temperature followed by the addition of 50 mL dichloromethane. The mixture was filtered, followed by evaporation of the solvent. Anhydrous ammonia was subsequently bubbled through the solution under stirring. The ligand, ammonium (O, O)-dialkyldithiophosphate, was then precipitated and recrystallized in benzene, washed with absolute ethyl ether, and dried in vacuum.

### Preparation and characterization of Cu nanoparticles

Cupric acetate (0.002 mol) was dissolved in 20 mL deionized water used as the precursor of Cu nanoparticles. A mixture of the ligand (O, O-di-*n*-cetyldithiophosphate) and sodium hypophosphite (NaH_2_PO_2_, 0.0015 mol) in 100 mL solvent of ethanol/water was stirred uniformly at 60°C. The solution of cupric acetate was introduced dropwise into the mixture, and the reaction system turned from colorless solution to yellow suspension. Then, the hydrazine solution (10 mL) was added to the mixture, and a dark colloid was observed. The mixture was stirred at 60°C for 0.5 h and then cooled to room temperature. The precipitate was separated by centrifugation and was washed subsequently with water and ethanol. After separation, the nanoparticles were dried in a vacuum oven at 45°C for 2 h.

The surface-modified Cu nanoparticles with various molar ratios of P to Cu (1:2, 1:5, and 1:10) were prepared by fixing the concentrations of copper salt and reductant, and varying the concentration of O, O-di-*n*-cetyldithiophosphate. Because the ligands act as particle protectors through coordinating the S-containing end groups on the copper particle surfaces and the hydrophobic carbon tails are pointed outward from the particles, the resulting copper nanoparticles with the modification layers should be hydrophobic and be dispersed in nonpolar solvents.

The phase properties of the surface-modified Cu nanoparticles were characterized by X-ray powder diffraction (XRD) using a Thermo X-ray diffractometer (Bruker, Germany) with monochromatized Cu Kα radiation (λ = 1.5405 Å). The differential scanning calorimeter (DSC/TG, NETZSCH STA 409 PC/PG) was used to analyze the thermal decomposition process of the particles with a heating rate of 10 K/min in N_2 _with a flow rate of 20 mL/min. Transmission electron microscopy (TEM) images were taken with JEM-200CX (JEOL, Japan) instrument using an operating voltage of 160 kV. Scanning electron microscopy (SEM) images were taken with field-emission scanning electron microscope, and the energy dispersive X-ray analysis (EDX) was carried out on the SEM equipped with energy-dispersive spectrometer (FEI SIRION-100, GENENIS-4000, Netherlands). A Nexus 470 Fourier transform infrared spectrometer (NICOLET, USA) was employed to observe the changes of organic functional groups.

### Preparation of nanofluids

Three types of nanofluids were prepared by dispersing different mass fractions of the surface-modified Cu nanoparticles in kerosene, toluene, and decahydronaphthalene as the base liquids without a dispersant. The samples were homogenized for about 5 min using an ultrasonic disrupter to ensure proper dispersion of the nanoparticles. The color of the suspension was observed to be puce.

### Measurements on viscosity and thermal conductivity

A capillary viscometer was utilized to determine the viscosities of the Cu nanofluids. The viscometer was filled with 15 mL nanofluid and was submerged into a thermostatic bath with a resolution of 0.01 K. The vertical angle of the viscometer was accurately controlled with a special tripod. The flow time was measured with a stopwatch to an accuracy of 0.01 s. The viscometer was calibrated with twice-distilled water. Each viscosity value of the nanofluid was reported by averaging over three consecutive runs. The flow time was reproducible to be ± 0.2 s and the uncertainty of viscosity was within ± 0.002 mPa s. The densities of all the nanofluids were measured by a 10-mL capillary-type pycnometer, which was calibrated with deionized double-distilled water. The dynamic viscosity, *η*, was calculated according to the equation:(1)

where *ρ *and *ν *are the density and kinematic viscosity of the nanofluid, respectively, at the same temperature.

Measurements of the thermal conductivities of Cu nanofluids were performed by means of a computer-controlled transient calorimeter [14]. The schematic diagram of the apparatus has been described previously in detail [15]. The nanofluid samples were added into the thermal conductivity cell, and a series of voltage differences (Δ*V*) of the unbalanced bridge were recorded with the time at each temperature. These data were utilized to calculate the slope of the voltage against time (d*V*/d*t*) of the unbalanced bridge. The thermal conductivities of the base fluids and nanofluids were calculated from the established equation between *λ *and d*V*/d*t*, and the enhanced ratios of thermal conductivity were then obtained. All the measurements were performed at atmospheric pressure.

### Thermal-oxidation tests

The Cu/kerosene-based nanofluids (0.1% Cu nanoparticles) were thermally oxidized in an isothermal apparatus. Each test tube containing 100-mL sample of Cu nanofluid was placed in the heated test well. The investigated samples were subjected to thermal oxidation at 120 or 140°C. The temperature remained steady within ± 1°C. The flow meters were employed to regulate the oxygen flow with the rate of 30 mL/min into each sample by means of a gas dispersion tube. A small number of aliquots (<0.5 mL) of the samples were removed from the test tubes at fixed time intervals for the hydroperoxide measurements. The hydroperoxides formed in the samples during the thermal oxidization process were determined through measuring the absorption spectra of the iodine-starch solutions using ultraviolet-visible spectrometry [16,17].

## Results and discussion

### Characterization of surface-modified Cu nanoparticles

**Figure 1:**
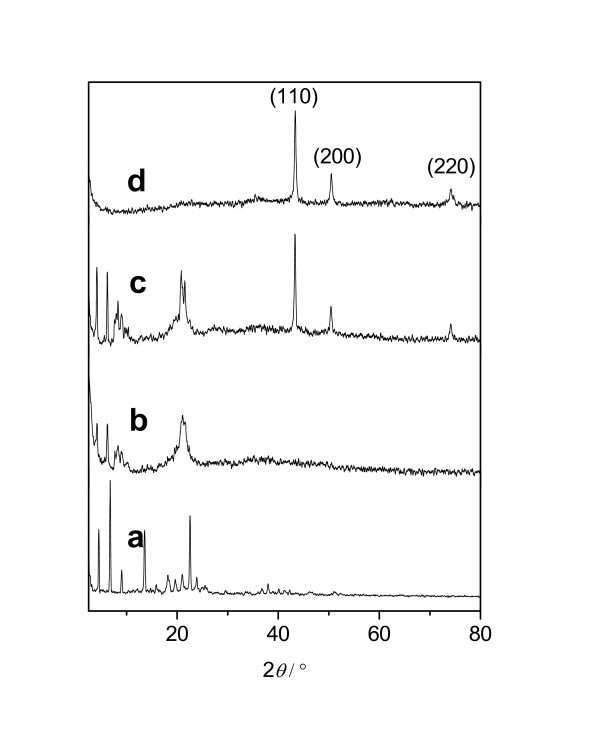
**XRD patterns of several samples**: (a) O, O-di-*n*-cetyldithiophosphate and surface-modified Cu products with molar ratios of P to Cu of (b) 1:2; (c) 1:5; and (d) 1:10.

Depending on the concentration of the ligand (O, O-di-*n*-cetyldithiophosphate), different generated products of surface-modified Cu nanoparticles have been obtained. The XRD patterns of several samples are shown in Figure 1. Figure 1a gives the powder XRD pattern of the O, O-di-*n*-cetyldithiophosphate. Figure 1b, c, d gives those of the products with molar ratios of P to Cu of 1:2, 1:5, and 1:10, respectively. The XRD pattern with P:Cu of 1:2 (Figure 1b) or 1:10 (Figure 1d) only exhibits the peaks of ligand or Cu, respectively. The XRD pattern shown in Figure 1c gives three characteristic peaks which can be indexed as face-centered cubic (*fcc*) structure Cu (111), (200), and (220). No visible XRD peaks arising from the impurity phase such as CuO and Cu_2_O are found. It is difficult for the formation of the core of Cu in the reaction solution when the ratio of ligand is too high. However, the ligand is not sufficient to modify the Cu particles produced in the reduction process, when the ratio of the ligand is too low. Therefore, the resultant product with P:Cu molar ratio of 1:5 is appropriate for preparing nanofluids. The characterizations and studies discussed in this section are focused on this composition.

**Figure 2:**
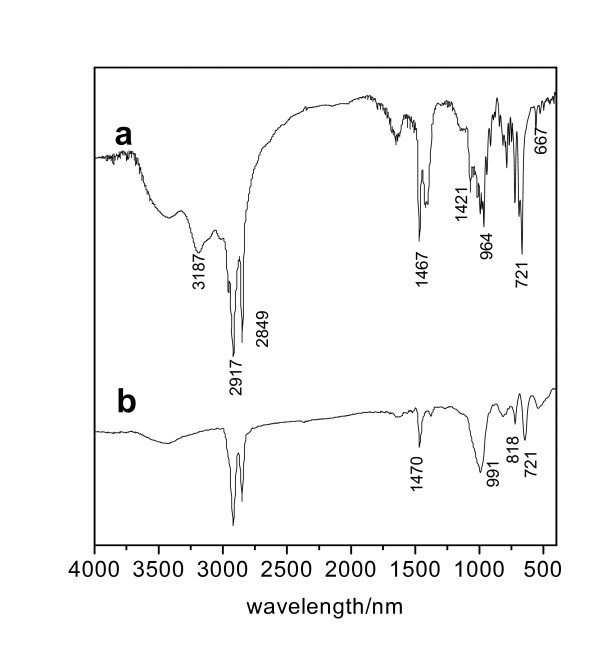
**Infrared spectra of (a) O, O-di-*n*-cetyldithiophosphate, and (b) surface-modified Cu nanoparticles**.

Infrared spectra of O, O-di-*n*-cetyldithiophosphate and surface-modified Cu nanoparticles are shown in Figure 2. As shown in Figure 2a, the absorptions at 2918 and 2850 cm^-1 ^are assigned to the stretching vibrations of CH_2 _groups, and the band at 1470 cm^-1 ^corresponds to the deformation vibration of CH_2 _groups. The absorption at 720 cm^-1 ^is due to the rocking vibration of the long chain alkanes [(CH_2_)_*n*_, *n *> 4]. The absorptions from 930 to 1050 cm^-1 ^are attributed to the stretching vibration of O-CH_2_. The absorptions at 687 and 670 cm^-1 ^are attributed to the stretching vibrations of P = S group, while the absorption at 582 cm^-1 ^is attributed to the stretching vibrations of P-S group. The absorption at 1400 cm^-1 ^is assigned to the stretching vibrations of NH_4_^+^. As shown in Figure 2b, the bands of C-H and O-CH_2 _are also observed in the surface-modified Cu nanoparticles, while the absorption peaks of P = S and P-S shifts, and the bands of N-H mostly disappear.

**Figure 3:**
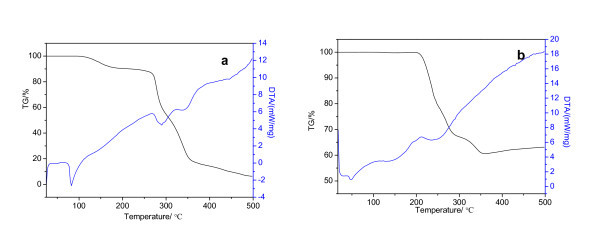
**TG/DTA curves**: **(a) **O, O-di-*n*-cetyldithiophosphate, and **(b) **surface-modified Cu nanoparticles.

Figure 3 shows the TG and DTA curves of O, O-di-*n*-cetyldithiophosphate and its surface-modified Cu nanoparticles, respectively. It is seen from the TG curve that O, O-di-*n*-cetyldithiophosphate and Cu nanoparticles begin to lose weight at 110 and 210°C, respectively. An obvious mass loss ranging from 210 to 350°C is observed for the Cu nanoparticles, and the total mass loss is about 40%. From the TG analyses, it can be concluded that the modification agent is coated on Cu nanocores through strong interaction, but not a mixture or simple absorption between Cu nanoparticles and modification agent. If the products comprise the mixture of Cu nanoparticles and modification agent, then the modification layers can be rinsed off in the synthesis proceeding, and very large amount of mass loss in the TG curve should not occur.

**Figure 4:**
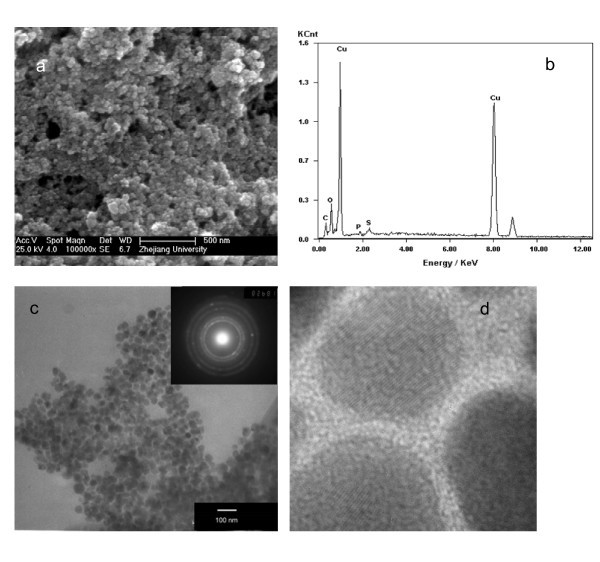
**(a) SEM image; (b) EDX spectrum of the surface-modified Cu nanoparticles; (c) TEM images SAED pattern; and (d) HTEM image**.

Figure 4 shows an SEM image (Figure 4a), an EDX spectrum (Figure 4b), a TEM image, and HTEM image of the surface-modified Cu nanoparticles. Nanoparticles with diameter in the range of 40-60 nm can be seen from the SEM image. The EDX analysis indicates that the Cu mass fraction in the prepared nanoparticles is 60-62%. This is consistent with the TG analysis. Figure 4c depicts a TEM image and the corresponding selected area electron diffraction (SAED) pattern. The micrograph reveals that the surface-modified Cu nanoparticles consist of spherical particles. The diffraction pattern further proves an *fcc *structure. The lattice fringes of Cu nanoparticles observed by close inspection with HRTEM are shown in Figure 4d.

The Cu nanoparticles are surface-modified by the organic ligands containing hydrocarbon tail. The coating layers should not easily separate from the surface of the Cu nanoparticles when the Cu nanoparticles are dispersed in the oil-based fluids. The lipophilic surface-modified Cu nanoparticles should be dispersed in hydrophobic solvents, such as toluene, chloroform, and liquid paraffin. It should not be dispersed in water and should not stay at the aqueous-organic interface. Therefore, the dispersion capability of Cu nanoparticles in hydrophobic solvents is improved by the surface modification, which enables the surface-modified Cu nanoparticles to be used as additives in oils.

### Viscosities and thermal conductivities of nanofluids

**Figure 5:**
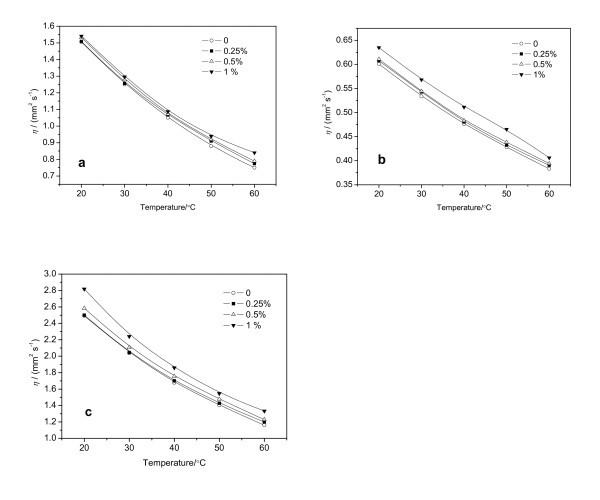
**Viscosities of Cu nanofluids**: **(a) **Cu/kerosene; **(b) **Cu/toluene; and **(c) **Cu/decahydronaphthalene.

The effects of both temperature and mass fraction of the nanoparticles on the viscosities of the nanofluids were investigated. Figure 5 shows the results of viscosity measurements for different fluid-based nanofluids at the temperature range from 20 to 60°C. The viscosity of a nanofluid decreases with increasing temperature, in a manner similar to that of a pure base liquid. It increases somewhat with increasing concentration of the nanoparticles. The addition of nanoparticles with 1% of mass fraction leads to no more than 5% increase of the viscosity. Therefore, the formation of nanofluids has no significant effect upon the viscous resistance.

**Figure 6:**
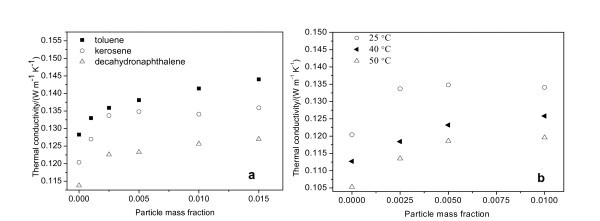
**Thermal conductivity of nanofluids**: **(a) **Variation of thermal conductivity of nanofluids at 25°C with mass fraction of nanoparticles; **(b) **variation of thermal conductivity with temperature for Cu/kerosene-based nanofluids.

Thermal conductivities of the nanofluids for different fluid-based nanofluids as a function of mass fraction of nanoparticles at 25°C are represented in Figure 6a. It can be seen that the thermal conductivity of Cu nanofluid increases with increasing mass fraction of nanoparticles for different fluid-based nanofluids. The relationship between the thermal conductivity enhancement and the mass fraction is nonlinear. The temperature effects on the enhancement of effective thermal conductivity are investigated by measuring the thermal conductivities of Cu/kerosene-based nanofluids at different temperatures, as shown in Figure 6b. It demonstrates that the thermal conductivities of the oil-based nanofluids increase clearly with the fluid temperature. The thermal conductivity of kerosene-based nanofluid increases by about 10, 13, and 14.6% with 1.0% (mass fraction) Cu nanoparticles at 25, 40, and 50°C, respectively. As the heat transfer in solid-liquid suspension occurs at the particle-fluid interface [18], an increase of the interfacial area can lead to efficient heat-transfer properties. Because the modified layers cap the copper cores and the metal surfaces do not directly contact with the base fluid, the surface-modified Cu nanoparticles are less effective than the uncoated Cu particles as far as the thermal-conductivity enhancement is concerned.

### Hydroperoxides in the Cu/kerosene-based nanofluids

**Figure 7:**
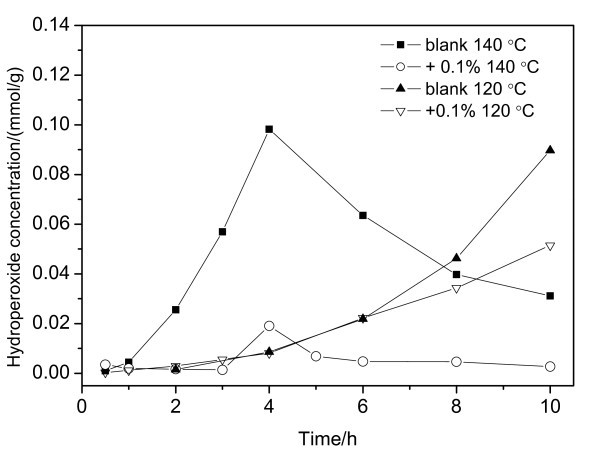
The change of hydroperoxide concentration in the nanofluid oxidized at 120°C and 140°C.

The hydroperoxides are the intermediates in the autoxidation reactions of hydrocarbon fuels. The hydroperoxide concentration is important for characterizing the thermal oxidation of a kerosene. Figure 7 gives the hydroperoxide concentration as a function of time in Cu/kerosene-based nanofluids and in kerosene without Cu nanoparticles thermal-oxidized at 120 and 140°C. As shown in Figure 7, the change of hydroperoxide concentration in the nanofluid oxidized at 120°C is nearly the same as that of the blank kerosene. At 140°C, the hydroperoxide concentrations in the nanofluid measured within 3 h are very low. It is clear that the hydroperoxide concentrations in the nanofluids are much lower than those in the blank kerosene during the thermal oxidation process. The Cu nanoparticles can significantly reduce the formation of the hydroperoxides in the kerosene. During the thermal oxidation at 140°C, the Cu nanoparticles deposit and react with oxygen. Therefore, the black CuO were found in the bottom of reactor. It indicated that the Cu nanoparticles were oxidized before the kerosene was oxidized. At lower temperatures, the coating layers on the surfaces of the nanoparticles prevent the Cu cores from oxidation. At higher temperatures, however, the coatings open or release from the surfaces, giving the opportunity for oxygen molecules to gain access to the Cu cores. The Cu nanoparticles then react with the oxygen before the kerosene is oxidized [19]. As a result, the hydroperoxide concentrations are observed to be relatively low in the Cu nanofluids. Appropriate amounts of metal nanoparticles added into a hydrocarbon fuel can enhance its thermal oxidation stability.

## Conclusions

The Cu oil-based nanofluids have been prepared by dispersing Cu nanoparticles modified with O, O-di-*n*-cetyldithiophosphate in kerosene, toluene, or decahydronaphthalene. The modified ligand is effective in improving the lipophilic property of Cu nanoparticles. The modified layers can be effectively coated on the surfaces of the Cu nanoparticles even when they are dispersed in the oil-based fluids. The thermal conductivity of nanofluids increases with the mass fraction of nanoparticles to some extent. The hydroperoxide concentrations are observed to be lower in the Cu nanofluids than in their base fluids. Appropriate amounts of metal nanoparticles added into a hydrocarbon fuel can enhance its thermal oxidation stability.

## Abbreviations

EDX: energy dispersive X-ray analysis; SAED: selected area electron diffraction; SEM: scanning electron microscopy; TEM: transmission electron microscopy; XRD: X-ray powder diffraction.

## Competing interests

The authors declare that they have no competing interests.

## Authors' contributions

DL: conceived of the study, carried out the experimental analyses, performed the XRD analyses, TEM characterizations and drafted the manuscript, WX: conceived the study, and participated in its design and coordination, WF: conceived the study, and participated in its design and coordination. All authors read and approved the final manuscript.
